# Assessment of the effect of platelet rich plasma on the healing of operated sacrococcygeal pilonidal sinus by lay-open technique: a randomized clinical trial

**DOI:** 10.1186/s12893-020-00865-x

**Published:** 2020-09-22

**Authors:** Mohamed M. Gohar, Reda F. Ali, Khaled A. Ismail, Taha A. Ismail, Nahla A. Nosair

**Affiliations:** 1 General Surgery Department, Kafr El Shiekh University Hospital, Kafr El Shiekh, Egypt; 2 Belbeis City, Egypt; 3 Clinical Pathology Department, Kafr El Shiekh University Hospital, Kafr El Shiekh, Egypt

**Keywords:** Pilonidal sinus, PRP, Lay-open excision

## Abstract

**Background:**

Sacrococcygeal pilonidal sinus disease (PSD) is an infection of the skin and subcutaneous tissue at the upper part of the natal cleft of the buttocks. Excision and healing by granulation “lay-open” method is still more preferable than other methods of midline closure or using flaps but the healing time is lengthy. The present study was performed to assess the healing promotion effect of platelet-rich plasma (PRP) on the pilonidal sinus wounds treated by the lay-open method.

**Methods:**

One hundred patients suffering from PSD were randomly divided into two groups, they were treated by the lay-open method, at General surgery department, Kafr El-Sheik University hospital, Egypt, during the period from December 2018 to December 2019. Group (A) was adopted the regular dressing postoperatively, while group (B) was treated with PRP injection into the wound at 4 and 12 postoperative days.

**Results:**

Accelerated rate of wound healing was detected in group (B) in day 10, with a significant difference detected in days 15, 20, 25 and 30 postoperative, with a mean time of complete healing 45 ± 2.6 days in group B, while it was 57 ± 2.4 days in group A with a *p*-value of 0.001 which indicates considerable effect in the treated group.

**Conclusions:**

PRP injection is an effective new technique in accelerating the healing of pilonidal wound after surgery, with a significant decrease in post-operative pain, complications and an early return to work.

**Trial registration:**

retrospectively registered. Trial registration number: 12/35/1016 issued on December 2018 from the Institution Review Board at Kafr El Sheikh University. ClinicalTrials.gov identifier: NCT04430413

## Background

Sacrococcygeal Pilonidal disease is an infection of the skin and subcutaneous tissue at the upper part of the natal cleft of the buttocks [1]. Pilonidal sinus disease (PSD) has an incidence of approximately 26 per 100,000 population with a male predominance of 2:1 and the mean age of those affected is from 19 to 30 years of age [2]. Individuals’ complaints are varied from asymptomatic midline pits in the natal cleft to symptomatic PSD with chronic discharge, pain and impact upon quality of life and social function [3]. The goals of treatment of PSD include eradication of the sinus tract, complete healing of the overlying skin, and prevention of recurrence. Many different techniques are available for surgical management of PSD [4]. Excision and healing by granulation is still preferred for low recurrence rate (3.4%) compared with other methods (20.6%) for midline closure and (10.3%) for flap closure, but healing time is lengthy and requires frequent daily dressing with a risk of infection and delayed wound healing [5]. Platelet Rich Plasma (PRP) which contains concentrated growth factors have been reported to accelerate wound healing by 30–40% giving a satisfactory outcome in the treatment of chronic skin and soft tissue lesions by supplying large amounts of growth factors and chemokines [6]. When platelets become activated, they secrete Seven fundamental protein growth factors initiating all wound healing process, including platelet-derived growth factor (PDGF), epidermal growth factor (EGF), transforming growth factor (TGF), vascular endothelial growth factor (VEGF), Fibroblast growth factor (FGF), connective tissue growth factor (CTGF) & insulin-like growth factor 1 (ILGF 1), which participate in the acceleration of wound-healing process [7–11]. Altogether, this study aims to evaluate the potential of PRP therapy for accelerating the healing of pilonidal sinus wounds.

## Methods

This is an open-label randomized clinical trial run for 1 year between December 2018 to December 2019.

### Ethics committee approval

A written informed consent was obtained from all patients. The research was conducted with approval from the institutional review board at Kafr El Shiekh University Hospital.

### Sampling

**Fig. 1:**
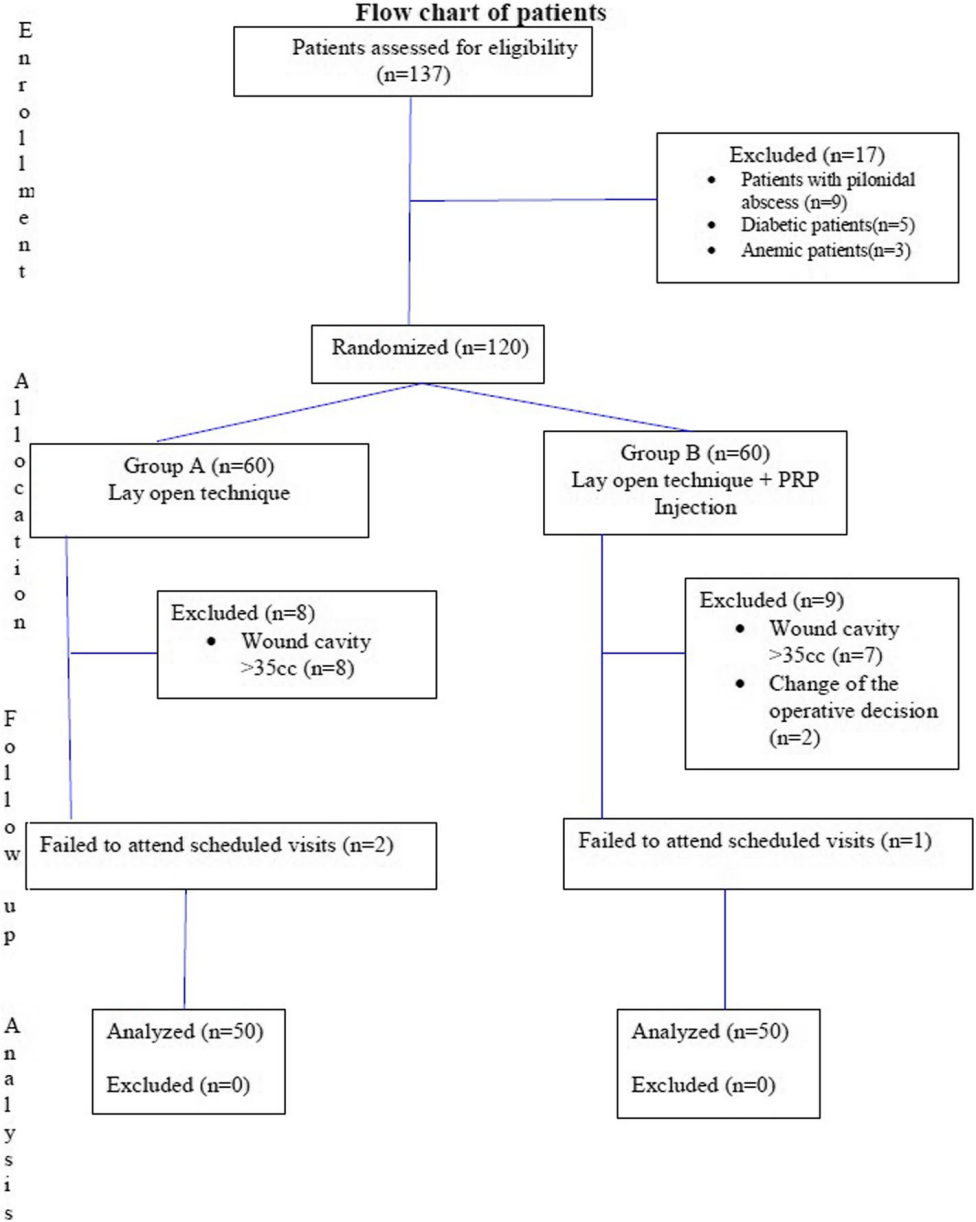
Flow chart of patients

One hundred patients were included, admitted to the General Surgery Department at Kafr El Shiekh University Hospital, Kafr El Shiekh, Egypt. This was an accessible sample of patients presenting to our department. They were divided randomly by closed envelope technique with those having the odd number allotted to group A and the even number allotted to group B. (Fig. 1).

Group A (control): included 50 patients that underwent a total excision of the pilonidal sinus adopting the lay-open technique and the wound was laid open for secondary healing.

Group B (PRP treated): included 50 patients that underwent the same procedure and on postoperative days 4 and 12 the platelet-rich plasma was injected into the surgical wound. Pits positions varied between each patients with the majority of pits lying in the midline and some patients had secondary pits outside the midline. No patients with pits below the coccyx were involved in the study as the wound volume would be more than 35 cc. 3 patients had recurrent sinus were included in the study.

### Inclusion criteria

All patients with PSD -including recurrent cases- who underwent Lay Open excision technique for PSD at Kafr El Shiekh University Hospital.

### Exclusion criteria

Patients with pilonidal abscess, diabetics, anemics, patients with Platelet count < 105/ul, patients on anticoagulant treatment, or had wound cavity > 35 cc were disqualified from this trial.

### Operative technique

The operation was done under spinal anesthesia in a prone position. A single dose of 3rd generation cephalosporin (1 g.) was given intravenously 30 min before surgery. The skin was disinfected with 10% povidone-iodine. The anal region was excluded from the operative field by drapes. Methylene blue dye was injected through the openings. An elliptical incision was made containing the sinus orifice and extended in-depth to the deep fascia. The diseased area was excised en-block. After hemostasis, the wound was filled with normal saline solution and then drained and the drained amount is measured to find the wound volume, the wound cavity > 35 cc was excluded from the study to ensure near-equal circumstances for comparison. The wound is then packed with the classic dressing of absorbent sterile cotton gauze.

### PRP preparation

PRP was prepared by the double Spain strategy. The patient was first sent to the university clinical pathology department where 25 cc of venous blood was acquired from the patient by venipuncture of the middle cubital lower arm vein. The blood was collected in seven 4 ml clean vacutainer tubes containing an anticoagulant Sodium Citrate 3.8%. The citrated blood was centrifuged at 1700 rpm for 15 min at room temperature, separating red blood cells at the bottom with plasma at the top and a “buffy coat” in between. The plasma and the buffy coat were aspirated from every test tube into a syringe and exchanged to another tube then centrifuged again at 3000 rpm for 10 min at room temperature. After the second centrifugation a two-section plasma was acquired: the upper part, comprising of platelet-poor plasma (PPP); and the lower part, comprising of platelet-rich plasma (PRP). The PPP was delicately aspirated, to separate it from the PRP.

### PRP injection method

Postoperative wound evaluation was done at the outpatient clinic and on the predetermined postoperative days, 4 and 12 PRP was injected in the wound through the granulation tissue beneath the skin to a 13 mm-depth of the wound using an insulin needle (0.1 cc/cm2) at each injection as fast as possible (the whole duration was less than 30 s), after that the wound was filled with the remaining PRP. Then, the surface was covered using sterile non-allergenic latex to avoid any PRP leakage for 24 h. After 24 h, the latex cover was removed and the usual dressing was performed.

### Postoperative care

All patients of both groups were discharged on the day of operation. Instructions for home dressing were given for each patient (wound irrigation with normal saline twice a day and classic dressing with absorbent sterile cotton gauze after disinfection with 10% povidone-iodine).

Ceftriaxone IM/24 h for 3 days was prescribed as a post-operative prophylaxis and paracetamol 500 mg tab triple-daily as pain killer with the avoidance of NSAID in order not to interfere with platelet function.

### Follow up

All patients were followed up regularly at the outpatient clinic in scheduled visits on days 4, 6, 9, 12, 17, 22, 27 postoperative and then every week (days 35, 42, 50 postoperative) till complete healing was achieved and Group B patients received the predetermined PRP injections in the wound on postoperative days 4 (first postoperative outpatient clinic visit and to ensure complete hemostasis of the wound) and day 12 (1 week later after the first PRP injection). During each visit, the assessment of the wound capacity and measurement of the pain score (Numeric rating scale from 1 to 10 was used to assess the severity of pain) was performed. Patients were assessed by the same surgeons in every visit.

### Parameters of evaluation

Patients were evaluated regarding post-operative healing; wound volume (primary outcome measure) (Cut off point was set to a certain volume of the wound upon which wound healing was assessed accordingly i.e. total time of healing is dependent on a specific volume capacity of the remaining gap after excision of the sinus and after this certain time, the healing would be called delayed wound healing) and post-operative pain duration (secondary outcome measure) on days 4, 12, 30 and 50 postoperative. Other measured data included the incidence of wound infection (surgical site infection), the duration of pain killer treatment and time to return to work. The wound volume was assessed by the same method used intra-operatively.

Data were analyzed using the SPSS software package version 20.0 (Prentice-Hall, Chicago, IL, USA). Qualitative data were described using the number and percent. Quantitative data were compared using the student t-test. A comparison between the two groups regarding categorical variables was done using the Chi-square (X2) test. When more than 20% of the cells have expected count less than 5, correction for Chi-square was conducted using Fisher Exact test or Monte Carlo correlation (MC). A “p” value of < 0.05 was considered to be statistically significant.

## Results

### Demographic data

Both groups were compared regarding the demographic data (Table 1). Clinical presentation and duration of symptoms are presented in Table 2 and Table 3. The number of pits was comparable in both groups ranging from 3 to 5 pits.

**Table 1:** Demographic findings among the studied group (mean and median are mentioned to summarize data)

Variable	Group A (***N*** = 15)	Group B (***n*** = 15)	***P***-value
**Sex (Frequency)**			
**Male**	43 (86.6%)	40 (80%)	
**Female**	7 (13.4%)	10 (20%)	
**Age** (**years)**			
**Mean ± SD**	26.27 ± 4.62	25.07 ± 4.83	1.3 (0.2) (NS)
**Median**	26	25	
**Range**	(18–35)	(18–35)

**Table 2:** clinical presentation before surgery

Symptoms	Group A (***N*** = 50)	Group B (***N*** = 50)	Chi square test ***p***-value
**Pain**	**17 (34.0%)**	**17 (34.0%)**	3.6 (0.16) NS
**Discharging sinus**	**27 (54.0%)**	**20 (40.0%)**	
**Pain and discharge**	**6 (12.0%)**	**13 (26.0%)**

**Table 3:** symptoms duration before surgery

Duration of symptoms (months)	Group A	Group B	***P***-value
**(mean ± SD)**	**9.33 ± 3.61**	**9 ± 3.94**	**0.8 (NS)**
**Range**	**5–17**	**5–18**	

### Postoperative follow-up

**Fig. 2:**
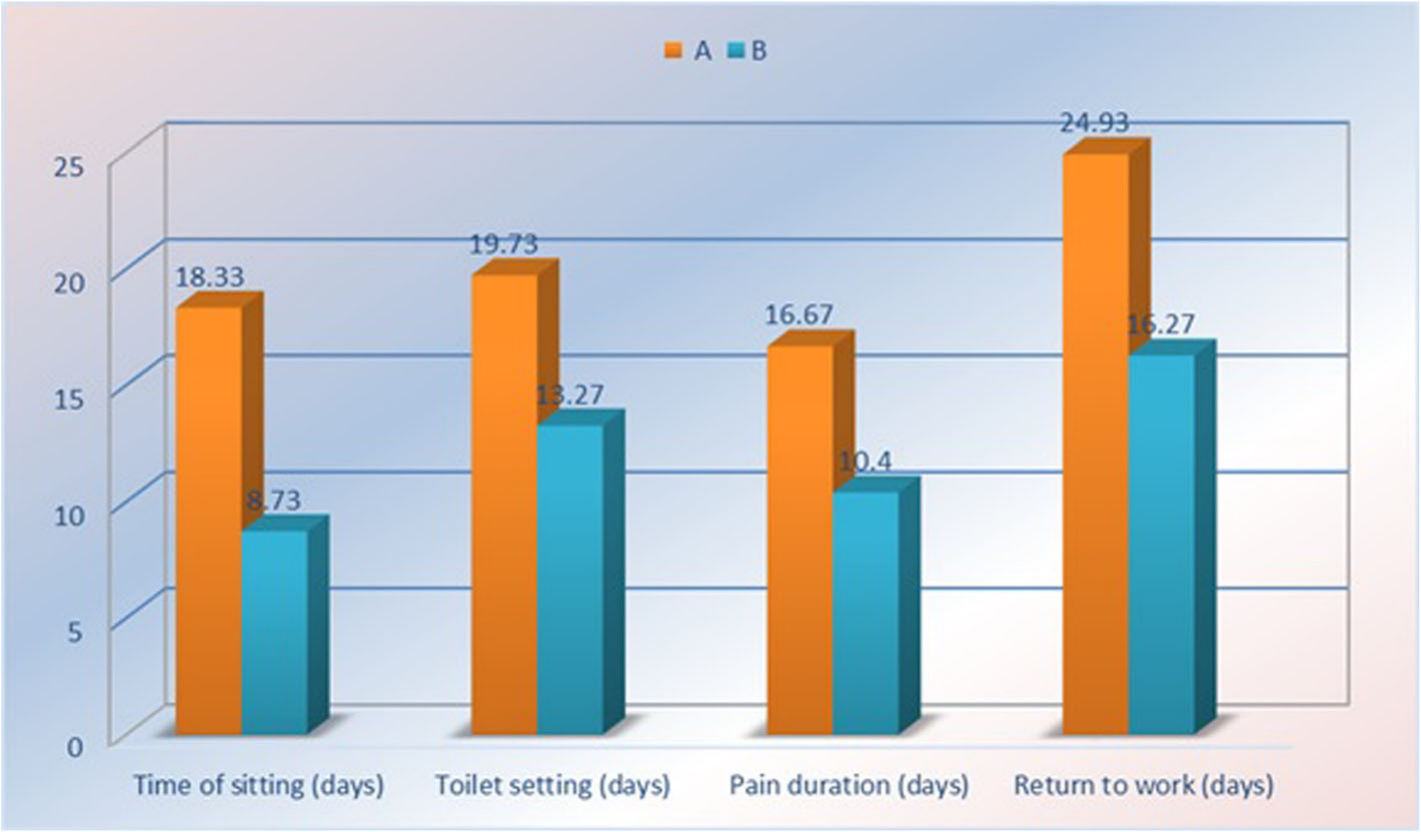
Postoperative outcome in both groups

In the postoperative period, there was a significant difference between group A and B regarding the time of sitting with a mean of (18.33 and 12.73) days, toilet sitting with a mean of (19.73 and 13.27) days, pain duration with a mean of (16.67 and 10.40) days and return to work with a mean of (24.93–16.27) days respectively as may be seen in (Table 4) (Fig. 2).

**Table 4:** Postoperative outcome in both groups regarding

Variable	Group A (***N*** = 50)	Group B (***N*** = 50)	T test p-value
**Time of comfort sitting (days)**	18.33 ± 2.41	12.73 ± 1.75	< 0.001** (HS)
**Toilet sitting (days)**	19.73 ± 2.60	13.27 ± 7.15	< 0.001** (HS)
**Pain duration (days)**	16.67 ± 1.83	10.40 ± 2.13	< 0.001** (HS)
**Return to work (days)**	24.93 ± 1.58	16.27 ± 2.25	< 0.001** (HS)

**Fig. 3:**
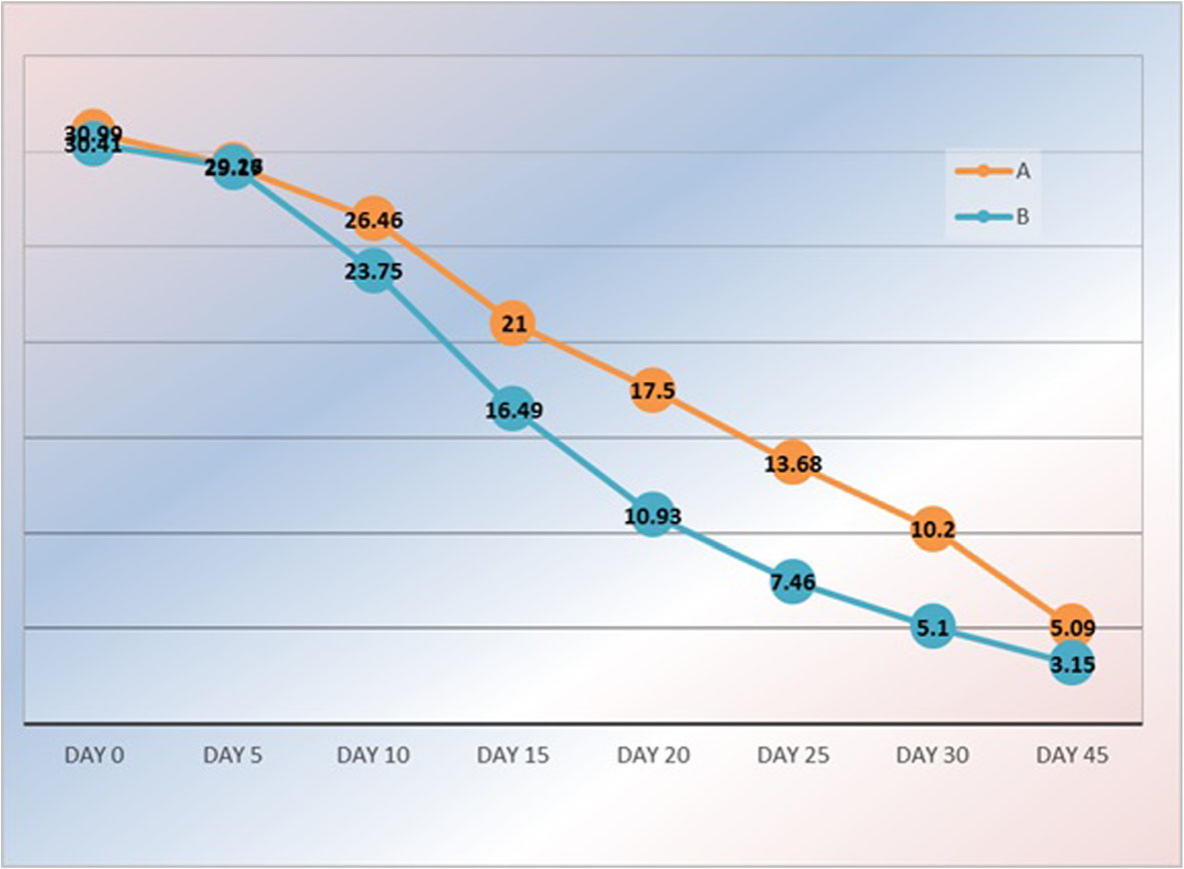
Mean wound volume in each group for the follow-up period

The rate of wound healing was estimated by wound volume (cm) starting from day 0. There was no significant difference in healing rate between both groups noted at days 5 and 10. But the start point of accelerated healing in the PRP group (B) was detected in day 10, with a highly significant difference detected in day 15, 20, 25 and 30, with a stationary course after day 30 till day 45, and showing no significant difference in wound volume between both groups in day 45 (Table 5). The mean time of complete healing was 45 ± 2.6 days in group B, while it was 57 ± 2.4 days in group A with a *p*-value of 0.001, with a statistically significant difference which indicates considerable effect in the treated group. (Fig. 3).

**Table 5:** postoperative follow up regarding wound volume (cm) in comparing with time (days)

Wound volume (cm)	Group A (***N*** = 50)	Group B (***N*** = 50)	***P***-value
**Day 0**	30.99 ± 2.37	30.41 ± 3.11	0.3 **(NS)**
**Day 5**	29.26 ± 1.76	29.17 ± 2.46	0.8 **(NS)**
**Day 10**	26.46 ± 0.97	22.75 ± 1.02	< 0.001** (HS)
**Day 15**	21.0 ± 1.0	16.49 ± 1.55	< 0.001** (HS)
**Day 20**	17.50 ± 1.25	10.93 ± 1.26	< 0.001** (HS)
**Day 25**	13.68 ± 0.88	7.46 ± 0.65	< 0.001** (HS)
**Day 30**	10.20 ± 0.30	4.49 ± 0.46	< 0.001** (HS)
**Day 45**	5.09 ± 0.38	3.15 ± 0.49	< 0.001** (HS)

### Postoperative complications

Wound infection and delayed wound healing which was found in 3 patients of group A (6%) (Complete healing was reached at the 75th postoperative day) and only one patient of group B (2%) (Complete healing was reached at the 60th postoperative day) with a significant difference (Table 6).

**Table 6:** postoperative complications. *Cut off point was set to a certain volume of the wound upon which wound healing was assessed accordingly

complication	Group A (***n*** = 50)	Group B (***n*** = 50)	Chi square (***P***-value)
**Wound infection**	10 (20%)	3 (6%)	4.3 (0.01)* S
Delayed wound healing*	3 (6%)	1 (2%)	0.3** NS

* Significant

** Not Significant

### CONSORT guidelines

The study was completely adherent to the CONSORT guidelines as published in 2010.

## Discussion

Platelet-rich plasma is a very rich platelet-derived autologous product. Platelet related products have been used in wound healing since 1985 with a satisfactory outcome so far in the treatment of chronic skin and soft tissue lesions, maxillofacial and plastic surgery wounds by providing large amounts of growth factors and chemokines. These growth factors act as a part of the cellular communication network that influences cell division, matrix synthesis and tissue differentiation [12].

This study was conducted to evaluate the potential of PRP in accelerating wound healing of those one hundred patients with sacrococcygeal pilonidal sinus who underwent lay-open excision of pilonidal sinus disease with secondary healing method which is an integral part of enhanced recovery program aiming at reducing morbidity and improving cosmesis.

Many different surgical techniques are recognized for the management of PSD. After excision, the wound may be left open to heal with granulation tissue or may be immediately closed by midline closure or by using a flap (Z-plasty, karydakis, or Rhomboid flap). Excision and healing by granulation is still preferred due to the low recurrence rate of (3.4%) compared with other methods (20.6%) for midline closure and (10.3%) for using flaps closure.4 As the operative time is about 25 min and the surgery is a day-case surgery, that makes the procedure less costly than the other techniques (where extra time and skill are needed for flaps placement as well as the need for drains, multi-drug usage, and more hospital stay).

The rate of wound healing and the time needed to reach the complete wound healing after operation remains the cornerstone of this study, the volume of the wound at day 0 (operation day) is nearly equal in both groups (30.99 ± 2.37) and (30.41 ± 3.11) in group A and B respectively with no significant difference (*p*-value 0.5). This starting point assures a fair comparison condition regarding the wound volume.

In group B the acceleration of wound healing was noted on day 10 with no significant difference (*p*-value =0.06) but was more obvious and remarkable on day 15 and 20 (*p*-value =0.001) this finding was reflected on the duration of pain (10.4 ± 2.31) days and the time of comfort sitting (12.73 ± 175) and the early return to work and normal daily activity (16.2 ± 2.27). Though the timing of the PRP injections were the same in all cases. Those findings are coming in accordance with the study of Spyridakis and colleagues (2009) [13] on 52 patients who underwent an open excision, divided into two groups 22 and 30 for the control and treatment group respectively.

The starting point of wound volume was similar to our study and accelerated wound healing was also noted in days 10 and 15.

Regarding postoperative complications, surgical site infection and delayed wound healing was noted in 3 patients in group A (6%) and one patient in group B (2%), while Baher and colleagues (2013) observed increase in the infection rate in treated group 4 patients (11%) in comparison with control group one patient (2.7%). They explained this finding by the possibility that PRP (mainly originate from blood and plasma) provides a favorable condition for bacterial growth [14]. Whereas, Spyridakis and colleagues (2009) [13] noticed the decrease in post-operative infected patients compared with the control group which was explained by the fact that platelets contain antimicrobial proteins that have bactericidal and fungicidal properties. In the case of infection ciprofloxacin and clindamycin were prescribed until control of infection also wound debridement was done whenever necessary to remove the necrotic tissues.

Our study limitation was the strict 1 year period during which only 100 patients were included in the study, more number of participants would have been more beneficial.

## Conclusion

PRP injection is an effective new technique in accelerating the healing of pilonidal wound after surgery, with a significant decrease in post-operative pain, complications and an early return to work compared to minimally invasive surgeries, flaps or endoscopic treatment.

## Supplementary information

**Additional file 1.**

## Data Availability

The datasets used and/or analysed during the current study are available from the corresponding author on reasonable request.

## Abbreviations

PSD: Pilonidal sinus disease
CONSORT: Consolidated Standards of Reporting Trials
