# Supplementary figures and images for: Assessment of the effect of platelet rich plasma on the healing of operated sacrococcygeal pilonidal sinus by lay-open technique: a randomized clinical trial
Source: BMC Surg. 2020 Sep 22;20:212. doi: 10.1186/s12893-020-00865-x (PMC7510145; doi:10.1186/s12893-020-00865-x)

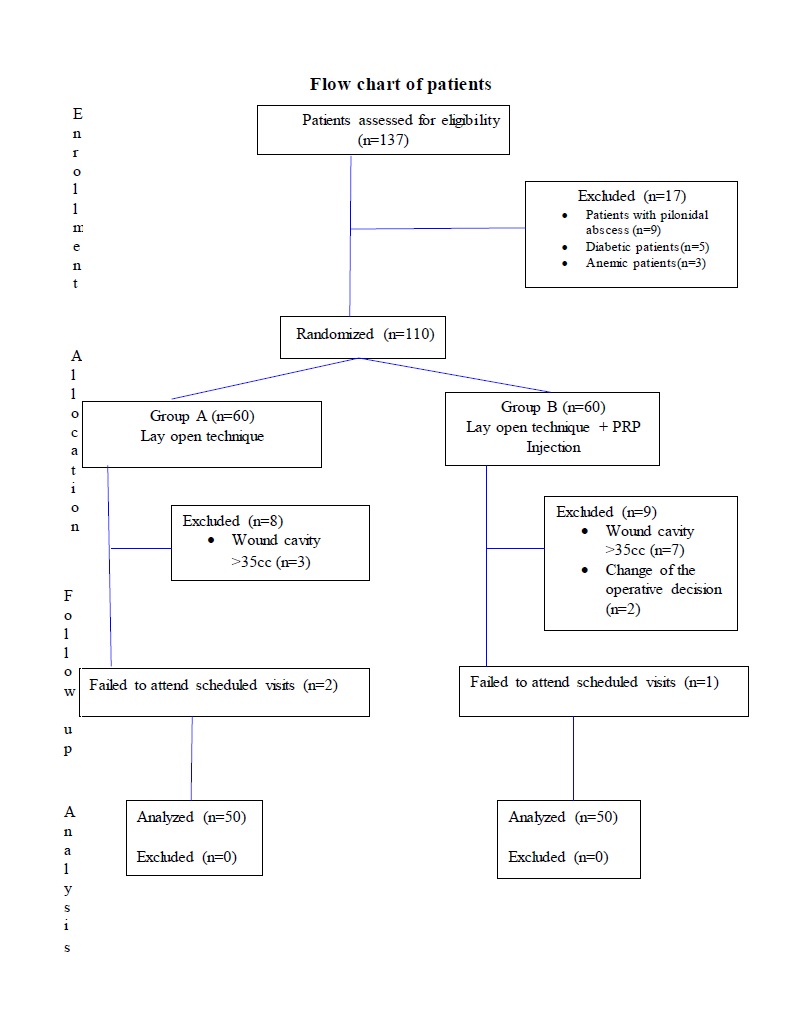

Supplement: Supplementary file 1 — Additional file 1. [file 12893_2020_865_MOESM1_ESM.jpg]
